# Aerobactin Synthesis Genes *iucA* and *iucC* Contribute to the Pathogenicity of Avian Pathogenic *Escherichia coli* O2 Strain E058

**DOI:** 10.1371/journal.pone.0057794

**Published:** 2013-02-27

**Authors:** Jielu Ling, Haizhu Pan, Qingqing Gao, Liping Xiong, Yefei Zhou, Debao Zhang, Song Gao, Xiufan Liu

**Affiliations:** Animal Infectious Disease Laboratory, Ministry of Agriculture, College of Veterinary Medicine, Yangzhou University, Yangzhou, Jiangsu, People's Republic of China; University of Rochester, United States of America

## Abstract

Aerobactin genes are known to be present in virulent strains and absent from avirulent strains, but contributions of *iucC* and *iucA*, which are involved in aerobactin synthesis, to the pathogenicity of avian pathogenic *Escherichia coli* (APEC) have not been clarified. In this study, effects of double mutants (*iucA*/*iutA* or *iucC*/*iutA*) compared to those of single mutants (*iucA*, *iucC* or *iutA*) of aerobactin genes on the virulence of APEC strain E058 were examined both *in vitro* (aerobactin production, ingestion into HD-11 cells, survival in chicken serum) and *in vivo* (competitive growth against parental strain, colonization and persistence). In competitive co-infection assays, compared to the E058 parental strain, the E058Δ*iucA* mutant was significantly reduced in the liver, kidney, spleen (all *P*<0.01), heart and lung (both *P*<0.001). The E058Δ*iutA* mutant also was significantly reduced in the liver, lung, kidney (all *P*<0.01), heart and spleen (both *P*<0.001). The E058Δ*iucC* mutant was significantly attenuated in the heart and kidney (both *P*<0.05) and showed a remarkable reduction in the liver, spleen and lung (*P*<0.01); meanwhile, both E058Δ*iucA*Δ*iutA* and E058Δ*iucC*Δ*iutA* double mutants were sharply reduced as well (*P*<0.001). In colonization and persistence assays, compared with E058, recovered colonies of E058Δ*iucA* were significantly reduced from the lung, liver, spleen and kidney (*P*<0.01) and significantly reduced in the heart (*P*<0.001). E058Δ*iutA* was significantly reduced from the heart, lung, liver, spleen and kidney (*P*<0.01). E058Δ*iucC*, E058Δ*iucA*Δ*iutA* and E058Δ*iucC*Δ*iutA* were significantly decreased in all organs tested (*P*<0.001). These results suggest that *iutA*, *iucA* and *iucC* play important roles in the pathogenicity of APEC E058.

## Introduction

Although iron is an essential element for all living cells, ferric iron is barely soluble at biological pH. Therefore, iron can be taken up in a complexed form via siderophores, a process that has been shown to contribute to the pathogenicity of avian pathogenic *Escherichia coli* (APEC) strains. The hydroxamate siderophore aerobactin has previously been associated with the pathogenicity of APEC strains [1], [2], [3], [4], [5], [6]. The ability of virulent microorganisms to grow under iron-limiting conditions may promote systemic infections [7], [8], [9]. As determined by the lethality for 1-day-old chicks in avian strains of *E. coli* [1], this physiological trait is highly correlated with APEC virulence. Lafont *et* *al.* stated that aerobactin genes are present in virulent strains but absent from avirulent strains, which has been confirmed by epidemiological studies [3]. Work undertaken by Tivendale *et* *al.* found that the *iucA* gene located on the pVM01 plasmid of APEC is highly correlated with virulence, adding support to the hypothesis that aerobactin is a virulence factor in APEC strains [10]. Dozois *et* *al.* revealed that *iutA* is dedicated to the virulence of APEC strain χ7122 [11]. In the APEC O2 strain, the aerobactin operon is situated on the pAPEC-O2-ColV plasmid [12], which contains genes responsible for the synthesis of the hydroxa siderophore aerobactin (*iucABCD*) and for ferric aerobactin uptake (*iutA*) [13], [14], [15]. Among APEC strains, iron acquisition systems can be encoded by plasmid genes [16], [17] or by chromosomal pathogenicity islands [18]. Aerobactin is a hydroxamate siderophore that is encoded by a plasmid operon [19], [20]. Hence, the expression of aerobactin should be associated with virulence of APEC. The aerobactin operon, which includes *iucABCD* and *iutA*, is localized to the APEC ColV plasmid [21].

Based on our previous observation that the *iucCD* gene of APEC E058 is up-regulated *in vivo* and the *iutA* transcription was also observed up-regulated in a microarray to detect the transcriptoms of avian pathogenic *Escherichia coli* (APEC) grown *in vivo* compared with those *in vitro* [22], we hypothesized that *iutA* or *iucC* and/or *iucD* is involved in the virulence of APEC E058. To our knowledge, the contribution of *iucC* to the pathogenesis of APEC and the effects of double mutations in *iucA/iutA* or *iucC/iutA* on APEC virulence compared to that of the each single mutation have not been reported. In this study, we chose to delete the single *iucC*, *iucA* or *iutA* gene or the double *iucC/iutA* or *iucA/iutA* genes to investigate their pathogenic roles in APEC O2 strain E058. A series of pathogenicity tests, including bactericidal assay in chicken serum, HD-11 cells ingestion assay, colonization and persistence in chickens, and *in vivo* competition assay, were employed to evaluate the pathogenicity of the APEC E058Δ*iucA*, E058Δ*iucC*, E058Δ*iutA*, E058Δ*iucA*Δ*iutA* and E058Δ*iucC*Δ*iutA* mutants compared with the E058 parent strain.

## Materials and Methods

### Ethics statement

This study was carried out in strict accordance with the recommendations of the Guide for the Care and Use of Laboratory Animals of the National Research Council. All experiments and procedures performed on the animals were approved by the Animal Care and Use Committee of Yangzhou University (approval ID: SYXK (Su) 2007–0005). Chickens were provided with food and water *ad libitum*. After 24 h of infection, the birds were euthanized by carbon dioxide asphyxiation and then dissected with aseptic surgical techniques.

### Bacterial strains, plasmids, primers, media and growth conditions

The strains, plasmids and cell line used in this study are listed in Table 1. The primers applied in this study are listed in Table 2. Cells were routinely grown at 37°C in Luria-Bertani (LB) broth or on LB agar with/without appropriate antibiotics: kanamycin (Km) (50 μg.ml^−1^), zeocin (25 μg.ml^−1^) or ampicillin (Amp) (60 μg.ml^−1^) unless otherwise specified. The chicken macrophage cell line HD-11 was maintained in Dulbecco's modified Eagle's medium (DMEM, Gibco, Grand Island, NY, USA) with 10% fetal bovine serum (FBS, PAA, Pasching, Australia) at 37°C, 5% CO_2_.

**Table 1:** Strains, plasmids and cell line used in this study.

	Description	Source
**Strains**		
E058	Wild-type avian *E. coli* serotype O2	[44]
DH5α	endA1 hsdR17 (r_k_^−^m_k_^+^)supE44 thi-1 recA1 gyrA (Nal^R^) RelA1Δ(lacIZYA-argF) U169deoR (φ80d lac Δ (lacZ) M15)	Invitrogen
LG1522	Aerobactin indicator strain	[5]
E058Δ*iutA*	*iutA* mutant of E058, Kan^r^	This study
E058Δ*iucA*	*iucA* mutant of E058, Zeo^r^	This study
E058Δ*iucA*Δ*iutA*	*iutA* and *iucA* double mutant of E058, Zeo^r^, Kan^r^	This study
E058Δ*iucC*	*iucC* mutant of E058, Zeo^r^	This study
E058Δ*iucC*Δ*iutA*	*iutA* and *iucC* double mutant of E058, Zeo^r^, Kan^r^	This study
Re-E058Δ*iucA*	Complementation of E058Δ*iucA*	This study
Re-E058Δ*iucC*	Complementation of E058Δ*iucC*	This study
**Plasmids**		
pMD®18-T simple vector	TA cloning vector, Ap^R^	Promega
pT-*iucA*	*iucA* cloned into pMD®18-T simple vector	This study
pT-*iucC*	*iucC* cloned into pMD®18-T simple vector	This study
pBluescript II SK(-)	Cloning vector	Fermentas
pS-*iucA*	*Hin*d III-*Bam*H I *iucA* fragment cloned into SK(-)	This study
pS-*iucC*	*Eco*R I-*Xba* I *iucC* fragment cloned into SK(-)	This study
pEM7/Zeo	Zeocin-resistant cassette	Invitrogen
pUC4K	Kanamycin-resistant cassette	Invitrogen
pS-*iucA*-Zeo	Zeocin-resistant gene inserted into pS-*iucA*	This study
pS-*iucC*-Zeo	Zeocin-resistant gene inserted into pS-*iucC*	This study
pGEX-6P-1	Bacterial expression vector	Amersham
pGEX-6P-1-*iucA*	*iucA* cloned into pGEX-6P-1 vector	This study
pGEX-6P-1-*iucC*	*iucC* cloned into pGEX-6P-1 vector	This study
**Cell line**		
HD-11	Chicken macrophage line, chicken myelomonocytic transformed by the myc-encoding MC29 virus	[45]

**Table 2:** Primers designed and used in this study.

Primer name	Sequence 5′–3′	Position (bp)	Source
iutA-F	CGAAGCTTTCTCAACCCACTGCTTCTT (*iutA* sense; *Hin*d III site underlined)	33–51	This study
iutA-R	TAGGATCCTGGTATAGCCATCGACCTT (*iutA* antisense; *Bam*H I site underlined)	1981–1999	This study
iucA-F	CTCAAGCTTAGTGCTTCCTGAATGCCT (*iucA* sense; *Hin*d III site underlined)	44–61	This study
iucA-R	CTCGGATCCTATGAGTCACCTGGTCAC (*iucA* antisense; *Bam*H I site underlined)	1556–1573	This study
pS-iucA-F	CTCGATATCTCAGGCAGGTATCGTTCA (pS-*iucA* sense; *Eco*R V site underlined)	492–509	This study
pS-iucA-R	CTCGAATTCCGATGCTTACTGTCAGCA (pS-*iucA* antisense; *Eco*R I site underlined)	1200–1217	This study
iucC-F	CTCGAATTCACTGGGATTTGGTCAACC (*iucC* sense; *Eco*R I site underlined)	14–31	This study
iucC-R	CTCTCTAGAATTCCTGAGTTACCAGCC (*iucC* antisense; *Xba* I site underlined)	1715–1732	This study
Zeo-BamH I-F	CTCGGATCCCACGTGTTGACAATTAAT (Inserting zeocin gene; BamH I site underlined)	1938–1955	This study
Zeo-BamH I-R	CTCGGATCCTCAGTCCTGCTCCTCGGC (Inserting zeocin gene; BamH I site underlined)	2366–2383	This study
GF	CACAGTCTTACTGCCAGT (*shiG* sense)	114–131	This study
GR	TTCTCGGTATCGGACAGA (*shiG* antisense)	266–283	This study
BF	TTGGTGAACAGCAATGGC (*iucB* sense)	701–718	This study
BR	ACCTCCGTGAAGAAGTGA (*iucB* antisense)	921–938	This study
DF	ATGGCATCACTGCCGATT (*iucD* sense)	782–799	This study
DR	TACGTGCAGATCTCCATG (*iucD* antisense)	1181–1198	This study
O2F	ATGTCGTGTTCCGTGCTCA (*O2ColV* sense)	1–19	This study
O2R	TCAGTAAGTTGGCAGCATC (*O2ColV* antisense)	204–222	This study
Re-iucA-F	TCAGGATCCATGATCCTGCCCTCTGAA (*iucA* sense; *Bam*H I site underlined)	1–18	This study
Re-iucA-R	TCAGAATTCTCAGACCTCCTGAGCCTG (*iucA* antisense; *Eco*R I site underlined)	1708–1725	This study
Re-iucC-F	CGCGAATTCATGAATCACAAAGACTGG (*iucC* sense; *EcoR* I site underlined)	1–18	This study
Re-iucC-R	GCGGTCGACTCATGATTCATATTCCTG (*iucC* antisense; *Sal* I site underlined)	1726–1743	This study

### DNA and genetic manipulations

All restriction and DNA-modifying enzymes and a 200 bp DNA marker purchased from Takara (Dalian, Liaoning, China) were used according to the supplier's recommendations. The Lamda DNA/*Eco*R I-plus-*Hin*d III molecular size standard was purchased from Fermentas (Shenzhen, Guangdong, China). Purification of PCR products and DNA fragments was performed using kits manufactured by Promega (Madison, WI, USA). The DIG High Prime DNA Labeling and Detection kit was purchased from Roche (Indianapolis, IN, USA). Transformation of *E. coli* strains was routinely carried out using electroporation. DNA and deduced amino acid sequence analyses were performed using DNASTAR Lasergene 8 software to predict conserved domains and using the search engine at http://blast.ncbi.nlm.nih.gov/Blast.cgi. DNA nucleotide sequences were determined by Sangon Co. LTD. (Shanghai, China). Reactions were carried out using *Taq* DNA polymerase (Fermentas) under the following conditions: 94°C for 4 min, followed by 30 cycles of 94°C for 1 min, 55°C for 1 min, and 72°C for 1 min and finally an extension at 72°C for 10 min.

### Construction of *iutA*, *iucA*, *iucC*, *iucA*/*iutA* and *iucC*/*iutA* mutant strains

The *iucA*, *iucC* and *iutA* genes were amplified from template which was a boiled preparation of the overnight culture of E058 using primer pairs iucA-F/iucA-R, iucC-F/iucC-R and iutA-F/iutA-R respectively (Table 2), and the PCR products were cloned into pBluescript SK (–) to form pS-*iucA*, pS-*iucC* and pS-*iutA* respectively. To form pS-*iucA*-Zeo, the *iucA* PCR product, which was amplified from pS-*iucA* using primers pS-iucA-F/R (Table 2) and digested with *Eco*R I-*Eco*R V, was ligated with the Zeo^r^ gene cassette originating from pEM7/Zeo digested with the same enzymes. To form pS-*iucC*-Zeo, the pS-*iucC* fragment digested with *Bam*H I was ligated with the Zeo^r^ gene cassette amplified from pEM7/Zeo using primers Zeo-BamH I-F/R and digested with *Bam*H I. To form pS-*iutA*-Kan, the pS-*iutA* plasmid and the pUC4K plasmid (containing the Kan^r^ gene) were both digested with *Pst* I and ligated together to form pS-*iutA*-Kan. Deletions of target genes *iucA*, *iucC*, *iutA*, *iucA/iutA* and *iucC/iutA* in the APEC O2 E058 strain essentially were performed as described by Xiong *et* *al.* [23].

### Complementation of mutants with the native *iucA* or *iucC* gene

For the complementation study, the native genes *iucA* (1725 bp) and *iucC* (1743 bp) were handled similarly by amplification using primers Re-iucA-F/R and Re-iucC-F/R (Table 2), with the introduced restriction enzyme recognition sites *Bam*H I-*Eco*R I and *Eco*R I-*Sal* I, respectively. To determine that the sequences were in frame, the *iucA* and *iucC* fragments were inserted into the pMD®18-T simple vector and sequenced by Sangon Co. LTD. (Shanghai, China). The *iucA* and *iucC* PCR products and expression vector pGEX-6P-1 were digested with *Bam*H I-*Eco*R I and *Eco*R I-*Sal* I, respectively, and ligated using T4 DNA ligase for 4 h at 22°C. The ligation mix was then transformed into DH5α and plated on LB agar plates containing ampicillin. Colonies were tested for the presence of *iucA* and *iucC* using standard primers iucA-F/iucA-R and iucC-F/iucC-R (Table 2), respectively. The modified plasmid pGEX-6p-1 with the *iucA* or *iucC* insert was isolated from DH5α and electroporated into E058Δ*iucA* or E058Δ*iucC* to complement the gene deleted.

### RT-PCR analysis

To extract total RNA from each strain, 1 ml of bacteria culture was collected by centrifugation. RNA was extracted by using the RNeasy Mini kit purchased from QIAGEN (Qiagen, Dusseldorf, Germany) and treated with an on-column Rnase-Free Dnase set. The first-strand synthesis of cDNA was primed with random primers using a high capacity cDNA archive kit (Applied Biosystems, Foster City, CA, USA). Primer sets for PCR amplification of target genes *shiG*, *iucA*, *iucB*, *iucC*, *iucD*, *iutA* and *O2-ColV16* in cDNA samples are listed in Table 2. In parallel, PCRs were performed with pAPEC-O2-ColV-like plasmid DNA as a positive control and cDNA samples without activation of the reverse-transcription (RT) as a negative control. The PCR products were resolved on 0.8% agarose gels and visualized by ethidium bromide staining.

### Growth in LB and under iron limitation

Each strain was cultured in LB medium with the appropriate antibiotic at 37°C overnight. The next day the cell density was estimated by spectrophotometry, and cultures were diluted in PBS prior to inoculation in LB medium with or without 200 µM 2,2′-dipyridyl (DIP) to achieve an approximate starting concentration of 10^3^ to 10^4^ colony-forming units (CFUs) per ml, which was confirmed by viable counts. Mixtures were incubated at 37°C with shaking, and aliquots of these cultures were removed at set time intervals for use in determining viable counts. The data represent averages of three independent assays.

### Bactericidal activity of specific-pathogen-free (SPF) chicken serum

The serum bactericidal assay was performed in a 96-well plate essentially as described previously [24] but with the following modifications. Briefly, complement-sufficient serum was prepared and pooled from SPF chickens (White Leghorn, Jinan SPAFAS poultry Co. LTD., Jinan, Shandong, China). The chicken serum was diluted to 0.5, 2.5, 5, 12.5 and 25% in pH 7.2 phosphate-buffered saline (PBS). Bacteria (10 μl containing 10^6^ CFU) were inoculated into reaction wells containing 190 μl of the diluted (25%) heat-inactivated SPF chicken serum or PBS alone and incubated at 37°C for 30 min. Serial dilutions (1∶10) of each well were plated onto LB agar plates. The resulting colonies were counted after 24 h of incubation.

### Animal infection models

A competitive co-infection model and a comparative single-strain infection model were used to investigate the contribution of the *iucA*, *iucC*, *iutA*, *iucA/iutA* and *iucC/iutA* genes to the virulence of APEC E058. Animals used in these studies were White Leghorn SPF chickens obtained from Jinan SPAFAS Poultry Co. LTD. The birds were treated in the experiments in accordance with the Regulations for the Administration of Affairs Concerning Experimental Animals (Approved by the State Council on October 31, 1988).

Briefly, for competitive co-infection assays, fifteen 3-week-old chickens were infected intratracheally with equal doses (1×10^7^ CFU each) of the wild-type strain E058 or the mutant strains E058Δ*iucA*, E058Δ*iucC*, E058Δ*iutA*, E058Δ*iucA*Δ*iutA* or E058Δ*iucC*Δ*iutA*. At 24 h post-infection, the spleen, heart, liver, lung and kidney of inoculated chickens were collected, weighed and homogenized, and serial dilutions were plated on LB medium with or without kanamycin or zeocin for selection of the mutant or total bacteria [25].

Animal experiments were also carried out to determine the colonization ability of the E058Δ*iucA*, E058Δ*iucC*, E058Δ*iutA*, E058Δ*iucA*Δ*iutA* and E058Δ*iucC*Δ*iutA* mutant strains in comparison with the E058 parent strain. For this single-strain infection model, 3-week-old White Leghorn chickens (15 chickens per group) were inoculated in the left thoracic air sac with 0.1 ml (10^7^ CFU) of a suspension containing the wild-type E058 strain or mutant derivatives. After 24 h, all chickens were euthanized and examined for macroscopic lesions. The heart, liver, spleen, lung and kidney of the chickens were collected, weighed, suspended in PBS and homogenized. Bacterial loads were determined by plating serial dilutions of the homogenates on selective LB agar medium [26].

### HD-11 ingestion assay

For ingestion assays, avian macrophage HD-11 cells were grown in DMEM with 10% FBS at 37°C with 5% CO_2_ at 2×10^5^ cells per well in 24-well cell culture plates and incubated for 24 h prior to ingestion assays. Bacteria were inoculated into cells at a multiplicity of infection (MOI) of 100. Inoculated cells were incubated at 37°C with 5% CO_2_ for 1 h under to allow the bacteria to be ingested by the cells. Thereafter, the cells were washed with PBS, and the extracellular bacteria were eliminated by culturing in DMEM medium containing gentamicin (100 µg/ml) at 37°C for 1.5 h prior to washing the cells again using PBS. The intracellular bacteria were treated with 1 ml 0.1% Triton X-100, and a 100 µl aliquot of this suspension was inoculated into 900 µl PBS. Serial dilutions (1∶10) of each sample were plated onto LB agar plates. The resulting colonies were counted after 24 h of incubation. Wells containing only HD-11 cells were used as negative controls. The assay was performed in triplicate. The ingestion ratio was determined by dividing the number of ingested bacteria by the number of bacteria in the initial inoculation.

### Aerobactin production

Mutants were also assessed for aerobactin production as described by Vidotto *et* *al*. [5]. Low-iron agar assay plates, composed of M-9 minimum salts, containing 200 µM 2,2′-dipyridyl and 0.2% glucose, were seeded with 1 ml/liter of an overnight culture of the indicator organism, *E. coli* LG1522, which is incapable of producing aerobactin but can use exogenously produced aerobactin. The E058 strain and its mutants were stab inoculated into the agar, and the plates were incubated at 37°C for 24 h. Following incubation, plates were observed for growth of the indicator organism around the stabs in a halo as evidence of aerobactin elaboration by the test mutants.

### Statistical analyses

Statistical analyses for *in vivo* tests were performed using GraphPad Prism v5.0 software package (GraphPad Software). The Wilcoxon matched-pair test was used to analyze data from the competition assay. The Mann Whitney U test was performed for analysis of results from the colonization and persistence assay.

## Results

### Construction of the *iutA*, *iucA* and *iucC* knockout mutants

The *iucA* and *iucC* genes situated on the pAPEC-O2-ColV-like plasmid from APEC E058 were sequenced and confirmed to be 1725 bp and 1743 bp in length, respectively. The putative *iutA* gene in the wild-type strain E058 was identified by BLAST searches of the *E. coli* plasmid pAPEC-O2-ColV complete sequence. Nucleotide sequence analysis showed that the *iutA* DNA fragment was 1966 bp in length. The APEC E058 mutants were created by the method described by Xiong *et* *al.* [23]. Sequence analysis revealed that the *shiG* gene encoding a conserved hypothetical protein is upstream of *iucA*. The *iucB* gene encoding the aerobactin biosynthesis protein IucB is downstream of *iucA* and upstream gene of *iucC*. The *iucD* gene, encoding the aerobactin IucD protein, is downstream of *iucC*. The *iutA* gene is downstream of *iucD* and upstream of the *O2-ColV16* gene.

The *iucA* and *iucC* knockout mutants were constructed by allelic exchange with an insertion of a Zeo^r^ cassette, while the *iutA* knockout mutant was made with an insertion of a Kan^r^ cassette. By sequence analysis of the PCR products, the *iucA* and *iucC* genes were confirmed to have been replaced by the Zeo^r^ gene in the pAPEC-O2-ColV-like plasmid of APEC E058 at the predicted position, and the generated mutants were named E058Δ*iucA* and E058Δ*iucC*, respectively. The *iutA* gene was also confirmed to have been replaced by the Kan^r^ gene in the pAPEC-O2-ColV-like plasmid of APEC E058 at the predicted position, and the generated mutant was named E058Δ*iutA*.

The *iucA* gene mutant plasmid containing the replacement Zeo^r^ gene was PCR amplified with primers iucA-F and iucA-R (Table 2) and purified for electroporation to E058Δ*iutA* competent cells as described previously [23]. After 18 h of incubation, the resulting Zeo^r^ Kan^r^ colonies were selected for PCR identification using primers iucA-F and iucA-R or iutA-F and iutA-R (Table 2) and analyzed by sequencing. The *iucA* and *iutA* double mutant was named E058Δ*iucA*Δ*iutA*. The *iucC* and *iutA* double mutant E058Δ*iucC*Δ*iutA* was generated using the same method.

### RT-PCR analysis

**Figure 1:**
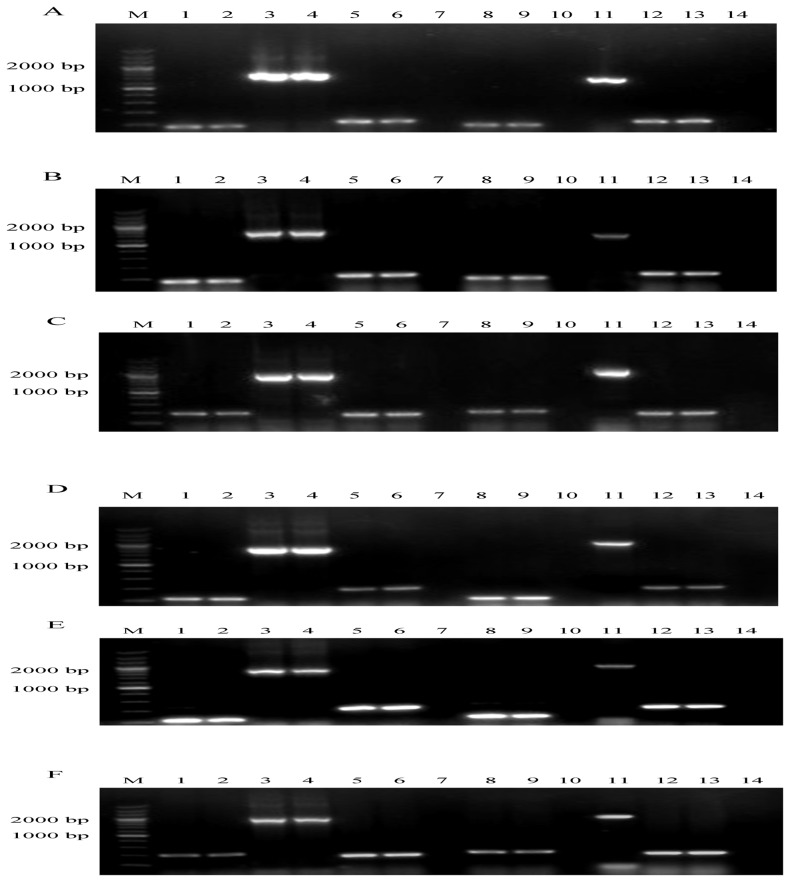
Detection of *iucA* (A, B), *iucC* (D, E) and *iutA* (C, F) gene expression by RT-PCR. (A). Detection of *iucA* gene transcription in E058 and E058Δ*iucA* by RT-PCR. Templates: lanes 1, 3, 5: cDNA derived from total RNA of E058. Lane 7: total RNA from E058 without activation of RT. Lanes 8, 10, 12: cDNA derived from total RNA of E058Δ*iucA*. Lane 14: total RNA from E058Δ*iucA* without activation of RT. Lanes 2, 4, 6: pAPEC-O2-ColV-like DNA from E058. Lanes 9, 11, 13: pAPEC-O2-ColV-like DNA from E058Δ*iucA*. Primers: Lanes 1, 2, 8, 9: GF/GR. Lanes 3, 4, 10, 11: iucA-F/iucA-R. Lanes 5, 6, 7, 12, 13, 14: BF/BR (Table 2). (B). Detection of *iucA* gene transcription in E058 and E058Δ*iucA*Δ*iutA* by RT-PCR. Templates: lanes 1, 3, 5: cDNA derived from total RNA of E058. Lane 7: total RNA from E058 without activation of RT. Lanes 8, 10, 12: cDNA derived from total RNA of E058Δ*iucA*Δ*iutA*. Lane 14: total RNA from E058Δ*iucA*Δ*iutA* without activation of RT. Lanes 2, 4, 6: pAPEC-O2-ColV-like DNA from E058. Lanes 9, 11, 13: pAPEC-O2-ColV-like DNA from E058Δ*iucA*Δ*iutA*. Primers: Lanes 1, 2, 8, 9: GF/GR. Lanes 3, 4, 10, 11: iucA-F/iucA-R. Lanes 5, 6, 7, 12, 13, 14: BF/BR (Table 2). (C). Detection of *iutA* gene transcription in E058 and E058Δ*iucA*Δ*iutA* by RT-PCR. Templates: lanes 1, 3, 5: cDNA derived from total RNA of E058. Lane 7: total RNA from E058 without activation of RT. Lanes 8, 10, 12: cDNA derived from total RNA of E058Δ*iucA*Δ*iutA*. Lane 14: total RNA from E058Δ*iucA*Δ*iutA* without activation of RT. Lanes 2, 4, 6: pAPEC-O2-ColV-like DNA from E058. Lanes 9, 11, 13: pAPEC-O2-ColV-like DNA from E058Δ*iucA*Δ*iutA*. Primers: Lanes 1, 2, 8, 9: DF/DR. Lanes 3, 4, 10, 11: iutA-F/iutA-R. Lanes 5, 6, 7, 12, 13, 14: O2F/O2R (Table 2). (D). Detection of *iucC* gene transcription in E058 and E058Δ*iucC* by RT-PCR. Templates: lanes 1, 3, 5: cDNA derived from total RNA of E058. Lane 7: total RNA from E058 without activation of RT. Lanes 8, 10, 12: cDNA derived from total RNA of E058Δ*iucC*. Lane 14: total RNA from E058Δ*iucC* without activation of RT. Lanes 2, 4, 6: pAPEC-O2-ColV-like DNA from E058. Lanes 9, 11, 13: pAPEC-O2-ColV-like DNA from E058Δ*iucC*. Primers: Lanes 1, 2, 8, 9: BF/BR. Lanes 3, 4, 10, 11: iucC-F/iucC-R. Lanes 5, 6, 7, 12, 13, 14: DF/DR (Table 2). (E). Detection of *iucC* gene transcription in E058 and E058Δ*iucC*Δ*iutA* by RT-PCR. Templates: lanes 1, 3, 5: cDNA derived from total RNA of E058. Lane 7: total RNA from E058 without activation of RT. Lanes 8, 10, 12: cDNA derived from total RNA of E058Δ*iucC*Δ*iutA*. Lane 14: total RNA from E058Δ*iucC*Δ*iutA* without activation of RT. Lanes 2, 4, 6: pAPEC-O2-ColV-like DNA from E058. Lanes 9, 11, 13: pAPEC-O2-ColV-like DNA from E058Δ*iucC*Δ*iutA*. Primers: Lanes 1, 2, 8, 9: BF/BR. Lanes 3, 4, 10, 11: iucC-F/iucC-R. Lanes 5, 6, 7, 12, 13, 14: DF/DR (Table 2). (F). Detection of *iutA* gene transcription in E058 and E058Δ*iucC*Δ*iutA* by RT-PCR. Templates: lanes 1, 3, 5: cDNA derived from total RNA of E058. Lane 7: total RNA from E058 without activation of RT. Lanes 8, 10, 12: cDNA derived from total RNA of E058Δ*iucC*Δ*iutA*. Lane 14: total RNA from E058Δ*iucC*Δ*iutA* without activation of RT. Lanes 2, 4, 6: pAPEC-O2-ColV-like DNA from E058. Lanes 9, 11, 13: pAPEC-O2-ColV-like DNA from E058Δ*iucC*Δ*iutA*. Primers: Lanes 1, 2, 8, 9: DF/DR. Lanes 3, 4, 10, 11: iutA-F/iutA-R. Lanes 5, 6, 7, 12, 13, 14: O2F/O2R (Table 2). A 200 bp marker (Takara) was used as the molecular size standard (M).

To determine whether the insertion had a polar effect on the upstream or downstream genes, total RNA samples extracted from the parental E058 and mutants E058Δ*iucA*, E058Δ*iucC*, E058Δ*iucA*Δ*iutA* and E058Δ*iucC*Δ*iutA* were analyzed by RT-PCR using primer sets designed for *shiG* [GF/GR], *iucA* [iucA-F/iucA-R], *iucB* [BF/BR], *iucC* [iucC-F/iucC-R], *iucD* [DF/DR], *iutA* [iutA-F/iutA-R] and *O2-ColV16* [O2F/O2R] (Table 2). When compared to the parental E058, the insertion of the Zeo^r^ gene in the E058Δ*iucA*, E058Δ*iucA*Δ*iutA*, E058Δ*iucC* or E058Δ*iucC*Δ*iutA* mutant only disrupted the transcription of the *iucA* or *iucC* gene (Figure 1A, 1B, lane10; 1D, 1E, lane 10). Meanwhile, insertion of the Kan^r^ gene in the E058Δ*iucA*Δ*iutA* or E058Δ*iucC*Δ*iutA* mutant only disrupted transcription of the *iutA* gene (Figure 1C, 1F, lane 10), and the upstream and downstream genes of all three target genes (*iucA*, *iucC*, *iutA*) were not influenced.

### Complementation studies and the result of aerobactin production

For complementation studies, the native *iucA* gene was amplified using primers Re-iucA-F/Re-iucA-R (Table 2) and subcloned into the pGEX-6P-1 vector by digestion with restriction enzymes *Bam*H I and *Eco*R I and ligation. The recombinant plasmid pGEX-6P-1-*iucA* was electroporated into E058Δ*iucA* to complement the deleted *iucA* gene, and colonies on LB agar containing ampicillin were tested for the presence of *iucA* using primers Re-iucA-F/Re-iucA-R (Table 2). A positive clone was named Re-E058Δ*iucA*. Using the same method, Re-E058Δ*iucC* was generated except that the restriction enzymes were *Eco*R I and *Sal* I. In testing for aerobactin production, the wild-type strain E058, the mutant E058Δ*iutA* and complementation strains Re-E058Δ*iucA* and Re-E058Δ*iucC* showed the ability to produce aerobactin, while the E058Δ*iucA*, E058Δ*iucA*Δ*iutA*, E058Δ*iucC* and E058Δ*iucC*Δ*iutA* mutants lost the ability to produce aerobactin (Data not shown).

### Characterization of the mutants

**Figure 2:**
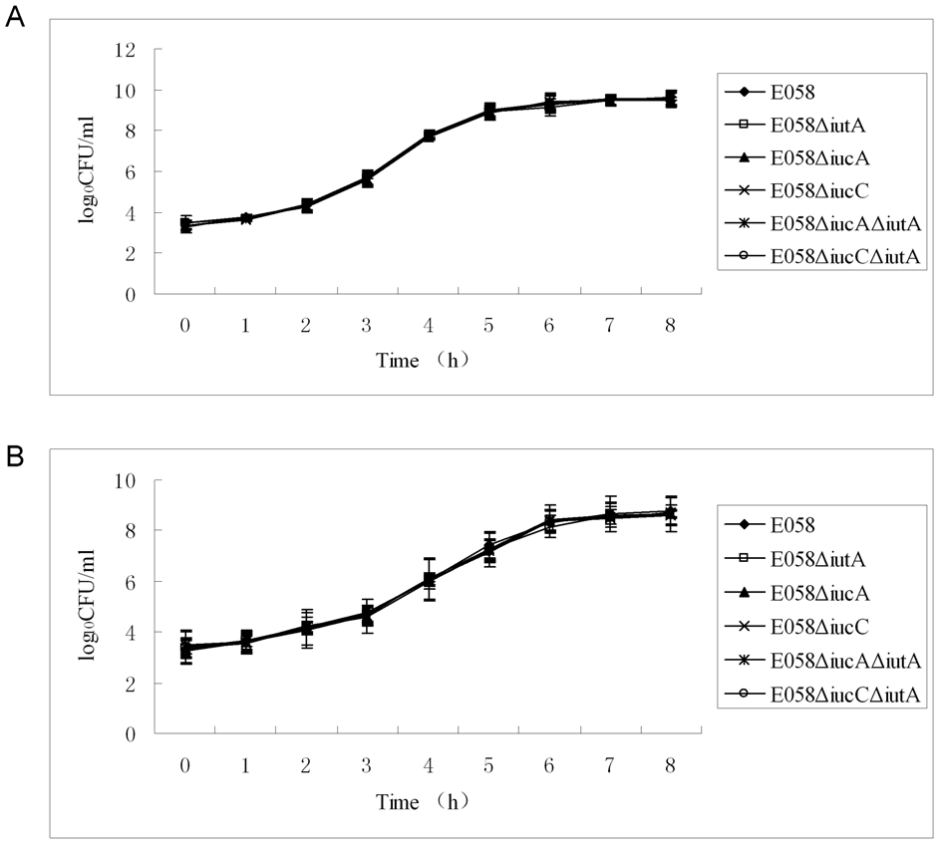
Growth curves of E058 wild-type strain and its mutants. (A) The E058(□), E058Δ*iutA*(▪), E058Δ*iucA*(▴), E058Δ*iucC*(×), E058Δ*iucA*Δ*iutA* (*) and E058Δ*iucC*Δ*iutA* (•) strains were grown in LB broth at 37°C, respectively, and their growth curves were determined by measuring viable counts (CFU ml^−1^). (B) Growth curves of the E058 and its mutants in LB containing 200 µM 2,2′-dipyridyl (DIP). The data represent averages of three independent assays.

**Figure 3:**
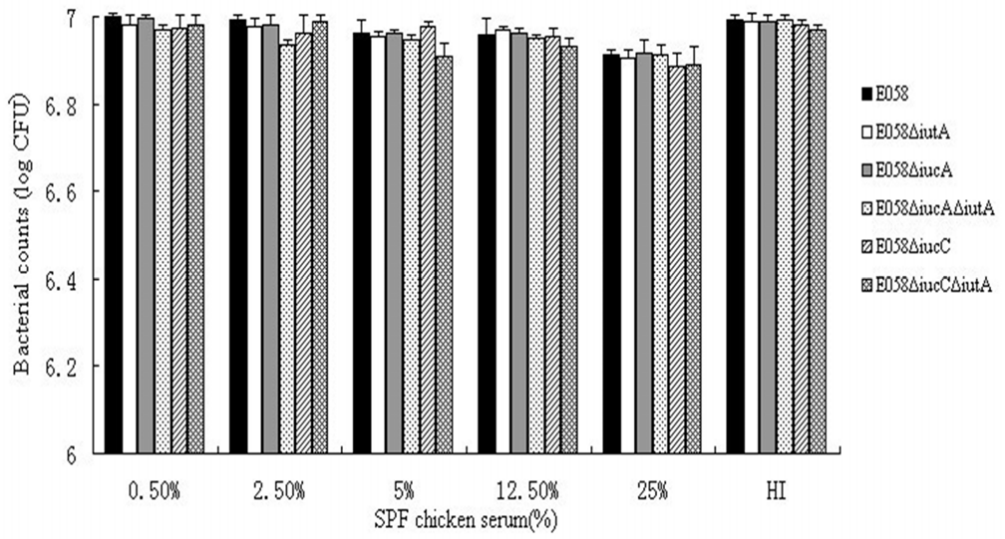
Bactericidal activity of SPF chicken serum against the wild type strain and mutants. Strains E058 (black bar), E058Δ*iutA* (white bar), E058Δ*iucA* (gray bar), E058Δ*iucA*Δ*iutA* (gray dots), E058Δ*iucC* (gray oblique lines), E058Δ*iucC*Δ*iutA* (gray grids). HI represents the group of heat-inactivated 25% SPF chicken serum used as controls for each strain tested. Data represent averages of three independent assays.

Growth curves, colonies, bactericidal assay and ingestion capability of the E058Δ*iucA*, E058Δ*iucC*, E058Δ*iutA*, E058Δ*iucA*Δ*iutA* and E058Δ*iucC*Δ*iutA* mutants were similar to those of the parental strain E058. Compared to the parent strain, no significant differences in the growth during the logarithmic phase were observed for all five mutants grown in LB rich medium and under iron limitation (Figure 2). Results of the bactericidal assay showed that the abilities of the E058Δ*iutA*, E058Δ*iucA*, E058Δ*iucA*Δ*iutA*, E058Δ*iucC* and E058Δ*iucC*Δ*iutA* mutants to survive in SPF chicken serum were not affected (Figure 3). The APEC E058 mutants (E058Δ*iutA*, E058Δ*iucA*, E058Δ*iucA*Δ*iutA*, E058Δ*iucC* and E058Δ*iucC*Δ*iutA*) had ingestion ratios (0.23%, 0.24%, 0.19%, 0.23% and 0.20%, respectively) which were not apparently different from that of the parental E058 (0.23%). Thus, the deletion of either the single gene of *iutA*, *iucA* or *iucC*, the double genes of *iucA*/*iutA* or *iucC/iutA* had no effect on the ingestion of APEC E058 into HD-11 cells.

### Deletions of *iutA*, *iucA*, *iucC*, *iucA*/*iutA* and *iucC*/*iutA* attenuate APEC E058 virulence *in vivo*

**Figure 4:**
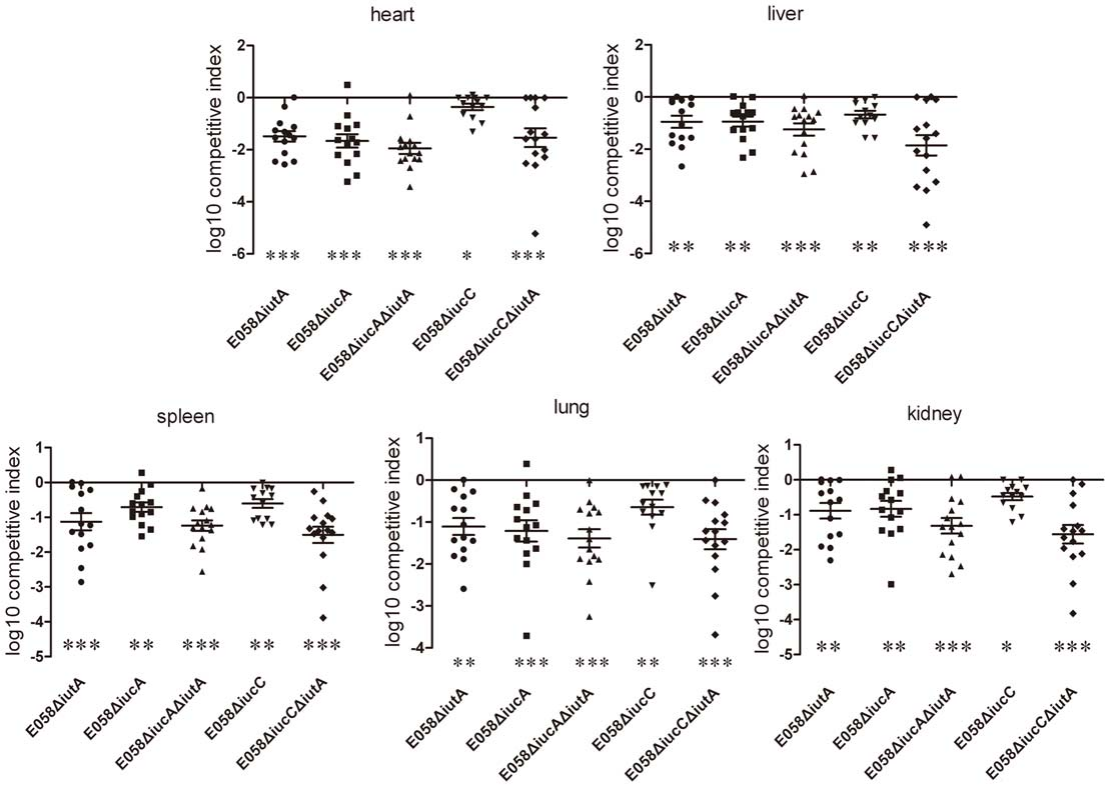
Competition assays between wild-type E058 and mutants E058Δ*iutA*, E058Δ*iucA*, E058Δ*iucC*, E058Δ*iucA*Δ*iutA* or E058Δ*iucC*Δ*iutA* inoculated simultaneously. Negative competitive index (CI) values indicate a decreased capacity of the mutants to compete for growth with the wild-type strain. Horizontal bars indicate the mean log_10_CI values. Each data point represents a sample from an individual chicken. Statistically significant differences in CI values between E058 and its mutants are indicated with asterisks (* *P*<0.05, ** *P*<0.01, *** *P*<0.001).

To determine the effects of *iutA*, *iucA*, *iucC*, *iucA/iutA* and *iucC/iutA* on the virulence of APEC E058 *in vivo*, competitive co-infections were carried out. As shown in Figure 4, at 24 h post-challenge, the E058Δ*iutA* mutant was significantly attenuated in the liver, lung and kidney (*P*<0.01) and was attenuated to an even greater degree in the heart and spleen (*P*<0.001) (Figure 4). The E058Δ*iucA* mutant was significantly attenuated in the liver, spleen and kidney (*P*<0.01), and especially in the heart and lung (*P*<0.001) (Figure 4). The E058Δ*iucC* mutant was also significantly attenuated in the heart and kidney (*P*<0.05) and exhibited more significant levels of attenuation in the liver, spleen and lung (*P*<0.01). Both double mutants (E058Δ*iucA*Δ*iutA* and E058Δ*iucC*Δ*iutA*) were significantly attenuated in all five tested tissues (*P*<0.001) (Figure 4).

**Figure 5:**
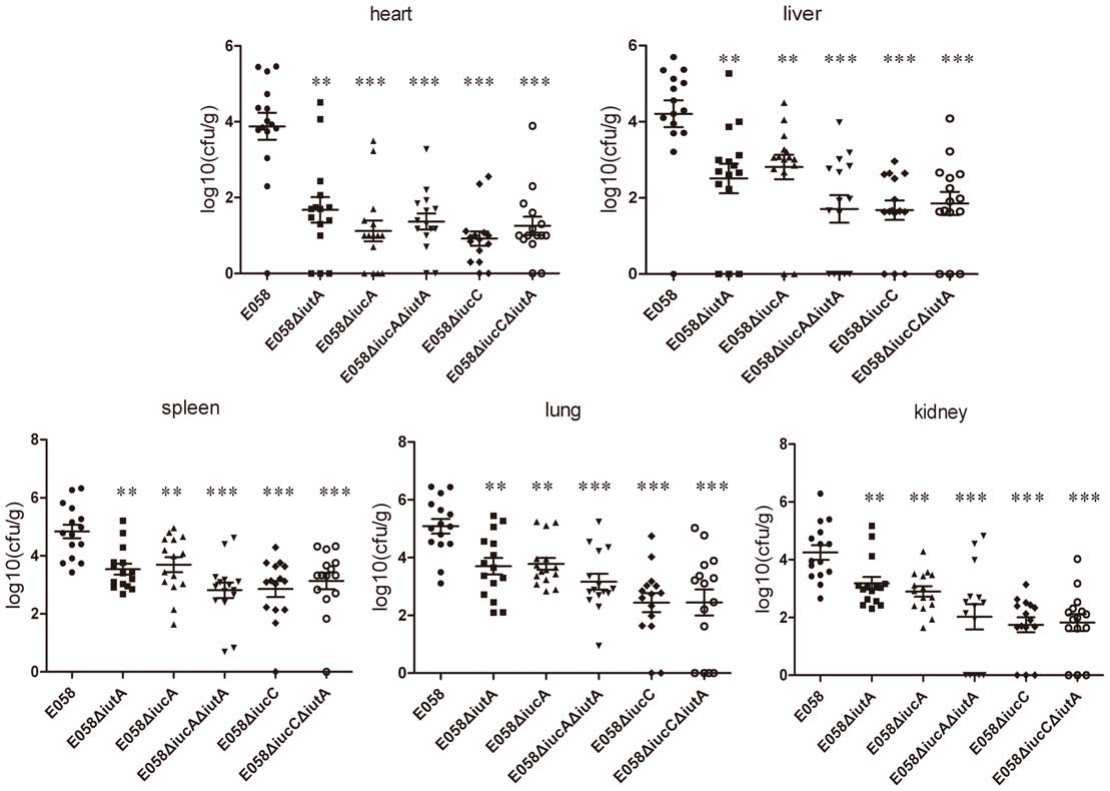
Colonization and persistence of wild-type strain E058 and its mutants E058Δ*iutA*, E058Δ*iucA*, E058Δ*iucC*, E058Δ*iucA*Δ*iutA* and E058Δ*iucC*Δ*iutA*. Data are presented as log_10_ CFU.g^−1^ of bacteria from tissues, and horizontal bars represent mean values. Each data point represents a sample from an individual chicken. Statistically significant differences are indicated with asterisks (** *P*<0.01, *** *P*<0.001), as determined by the Mann Whitney U test.

When birds were infected with the E058Δ*iutA*, E058Δ*iucA*, E058Δ*iucA*Δ*iutA*, E058Δ*iucC* and E058Δ*iucC*Δ*iutA* mutants, distinct reductions in the number of bacteria recovered from all tested tissues were observed compared to those of birds infected with E058. At 24 h after infection with the E058 parent strain, maximum colonization was observed in the lung (1.1×10^5^ CFU.g^−1^), and minimal colonies were obtained in the heart (8.7×10^3^ CFU.g^−1^), while colony numbers in the spleen (6.3×10^4^ CFU.g^−1^), liver (2.1×10^4^ CFU.g^−1^) and kidney (3.2×10^4^ CFU.g^−1^) were intermediate (Figure 5). Compared to the E058 wild-type strain, the mutant E058Δ*iutA* colony numbers were significantly decreased in the lung (6.5×10^3^ CFU.g^−1^) (*P*<0.01), heart (70 CFU.g^−1^) (p<0.01), kidney (2.6×10^3^ CFU.g^−1^) (p<0.01), liver (5.88×10^2^ CFU.g^−1^) (p<0.01) and spleen (5.9×10^3^ CFU.g^−1^) (*P*<0.01) and colonization with the E058Δ*iucA* mutant was significantly lower in the lung (7.8×10^3^ CFU.g^−1^) (*P*<0.01), spleen (6.8×10^3^ CFU.g^−1^) (*P*<0.01), heart (12 CFU.g^−1^) (*P*<0.001), liver (8.1×10^2^ CFU.g^−1^) (*P*<0.01) and kidney (9.0×10^2^ CFU.g^−1^) (*P*<0.01) (Figure 5). Levels of the E058Δ*iucA*Δ*iutA* mutant colonized in the lung (3.6×10^3^ CFU.g^−1^), spleen (1.02×10^3^ CFU.g^−1^), heart (14 CFU.g^−1^), liver (82 CFU.g^−1^) and kidney (96 CFU.g^−1^) were sharply decreased compared to those of the E058 parent strain (*P*<0.001) (Figure 5). Interestingly, numbers of the E058Δ*iucC* mutant in the lung (4.3×10^2^ CFU.g^−1^), spleen (8.6×10^2^ CFU.g^−1^), heart (9 CFU.g^−1^), liver (67 CFU.g^−1^) and kidney (64 CFU.g^−1^) also dropped dramatically compared to those of E058 (*P*<0.001) (Figure 5). Not surprisingly, the E058Δ*iucC*Δ*iutA* mutant colonized in the lung (4.2×10^2^ CFU.g^−1^), spleen (1.3×10^3^ CFU.g^−1^), heart (17 CFU.g^−1^), liver (90 CFU.g^−1^) and kidney (93 CFU.g^−1^) also decreased remarkably compared to E058 (*P*<0.001) (Figure 5). On average the mutant strains were reisolated at levels of 10–1000 times lower than the wild-type strain from the internal organs tested at 24 h post-challenge. These results indicated that *iutA* and both *iucA* and *iucC* genes are involved in the process of systemic infection of APEC E058.

## Discussion

Many types of bacteria have several iron uptake systems to survive and grow in different host environments [26]. In the virulence assay, the mutants were significantly attenuated in the observed tissues. Sequestration of iron results in an extremely low free iron concentration that would limit bacterial growth in this complicated environment *in vivo* [25]. The ability to obtain iron from the host determines the pathogenicity of APEC strains and may be increased by the aerobactin and iron system [27].

Iron has many vital functions in bacteria and is a cofactor for a large number of enzymes [28]. The enterochelin system is one of the iron uptake systems that is chromosomally encoded and appears less able to compete with transferrin *in vivo*, although it is very efficient *in vitro* [29]. By contrast, the aerobactin system is considered to be efficient *in vivo* and is involved in the invasive properties of human enteroinvasive *E. coli* and *Shigella* species [30], [31]. The aerobactin operon encoded by either plasmids or chromosomes in several species of enteric organisms [6], [15], [20], [31], [32], [33], [34], [35] contains genes responsible for the synthesis of the hydroxamate siderophore aerobactin (*iuc*) and for ferric aerobactin uptake (*iut*) [13], [14], [35]. Both components are strongly induced under iron starvation. Because aerobactin genes are present in virulent strains and absent from avirulent strains [3], they are regarded to play independent roles in the pathogenicity of APEC, especially in deep tissue damage, and its persistent infection [11]. The five genes encoding aerobactin are localized to an operon, which is present in the ColV plasmid [17]. The *iucC* gene is related to the aerobactin synthetase reaction, whereas the deletion of *iucA* will result in a severe polar effect [36]. A previous study from our laboratory [23] and independent reports from others [21], [37] have provided a general overview of the aerobactin operon and several iron uptake system [38], [39], [40], [41]. To date, many studies have focused on the characterization of the *iuc* genes [29], [35], [36], [42], but the relationship between the *iucC* gene and the pathogenicity of APEC has not been clarified.

In this study, the E058Δ*iucA*, E058Δ*iucC*, E058Δ*iucA*Δ*iutA* and E058Δ*iucC*Δ*iutA* mutants with deletions of the *iucA*, *iucC*, *iucA/iutA* and *iucC/iutA* genes, respectively, lost the capacity to produce aerobactin, while the complementation of E058Δ*iucA* and E058Δ*iucC* restored their ability to produce aerobactin similar to the E058 parent strain. Meanwhile, the mutant E058Δ*iutA* also showed the capacity to produce aerobactin. In the acquisition of iron, APEC O2 is known to contain at least two other chromosomal operons (yersinabactin and enterobactin) [17]. Although we deleted the aerobactin receptor gene *iutA*, the other two chromosomal operons (yersinabactin and enterobactin) may have helped to compensate for the loss of *iutA*. In addition, *iucA* or *iucC* is responsible for the synthesis of the hydroxa siderophore aerobactin. While the deletion of one or both of these two genes may have resulted in the failure to produce aerobactin (hydroxamate type siderophore), it may not have influenced the other two chromosomal operons (yersinabactin and enterobactin) in the acquisition of iron.

In LB broth and under conditions with limited iron, there was no obvious difference in the growth of the mutants and wild-type parent. During the assays of bacterial ingestion into HD-11 cells, no obvious difference was observed between the wild-type E058 and its mutants. These findings correspond to results of a previous study on E058Δ*iucB* and E058Δ*iucB*Δ*iutA* [23].

In the single-strain infection model, compared to E058, colonization levels of the E058Δ*iucA* and E058Δ*iutA* mutants were reduced in all five tested tissues (*P*<0.01 or *P*<0.001), and that of the E058Δ*iucC* mutant was remarkably reduced in the above-mentioned tissues (*P*<0.001). The same results were observed for both the E058Δ*iucA*Δ*iutA* and E058Δ*iucC*Δ*iutA* double mutants.

The capacity of the E058Δ*iutA*, E058Δ*iucA*, E058Δ*iucA*Δ*iutA*, E058Δ*iucC* and E058Δ*iucC*Δ*iutA* mutants to compete for growth in 3-week-old SPF chicken tissues with the E058 wild-type strain was also evaluated in a co-infection model. Compared to its parental strain, the E058Δ*iutA* mutant showed a significant reduction of bacterial counts in the heart, liver, kidney, spleen and lung (*P*<0.05 or *P*<0.01). The E058Δ*iucA* mutant showed a remarkable reduction in all the tissues mentioned above (*P*<0.01 or *P*<0.001). The E058Δ*iucC* mutant was also significantly attenuated in all the tissues mentioned above (*P*<0.05 or *P*<0.01). Furthermore, highly significant reductions of both the E058Δ*iucA*Δ*iutA* and E058Δ*iucC*Δ*iutA* double mutants were observed in all five tested tissues (*P*<0.001). The co-infection assay demonstrated that the deletion of either the *iutA*, *iucA* or *iucC* gene reduced the virulence of the APEC strain E058. However, it seemed a greater degree of colonization defects in an independent challenge versus a co-challenge experiment. As the individual infection results showed a 2-log decrease in colonization for most of the mutants in most tissues (10–1000-fold). Whereas, the co-challenge experiment results showed a 1-log fitness defect for most mutants in all tissues. These results implied that the exogenous siderophores synthesized by the wild-type strain may be to a certain extent complement the effect of the siderophore synthesis mutations in a co-infection model. Similarly, a *ΔiucBΔentD* double mutant, defective in synthesis of both siderophores, was rescued by co-infection with a wild-type strain in the mouse UTI model [43].

The pathogenicity of mutants with deletion of either the whole *iucABCDiutA* operon or the single gene *iutA* in APEC χ7122 is attenuated [11]. Meanwhile, the effect of the single gene *iucA* or *iucC*, and the double genes *iucAiutA* or *iucCiutA* on the virulence of APEC remains to be determined. Tivendale *et* *al*. demonstrated that the *iucA* gene could be amplified only from the three most virulent strains, E3, E30 and E956, but not from the less virulent strains, E133, E1043 and E1292. As discussed by these authors, the identification of potential virulence genes in avian colibacillosis can be attempted by using different approaches, such as to generate isogenic mutants of these genes and determine their virulence in birds [10]. In this study, we used this approach to explore the effects of *iucA* and *iucC* genes on the pathogenicity of the APEC strain E058. In a previous study, we demonstrated that either the *iucB* gene or *iutA* gene is likely involved directly or indirectly in iron uptake, with no obvious synergistic effect between these two genes, as it relates to the pathogenicity of APEC E058 [23]. To our knowledge, the present study is the first to report the contribution of *iucC* to the pathogenesis of APEC and the effects of double mutations in *iucA*/*iutA* or *iucC*/*iutA* on APEC virulence compared to that of each simple mutation. Taken together, the above results demonstrate that the *iucA* and *iucC* genes, which participate in aerobactin synthesis, play an important role in the pathogenicity of the APEC strain E058.
